# Organically surface engineered mesoporous silica nanoparticles control the release of quercetin by pH stimuli

**DOI:** 10.1038/s41598-022-25095-4

**Published:** 2022-11-30

**Authors:** Ozi Adi Saputra, Windy Ayu Lestari, Viardi Kurniansyah, Witri Wahyu Lestari, Takashi Sugiura, Rino R. Mukti, Ronny Martien, Fajar Rakhman Wibowo

**Affiliations:** 1 Master Program of Chemistry, Faculty of Mathematics and Natural Sciences, Universitas Sebelas Maret, Jl. Ir. Sutami 36A, Surakarta, 57126 Indonesia; 2 Department of Chemical Engineering, College of Engineering, National Taiwan University, No. 1, Section 4, Roosevelt Rd, Da’an District, Taipei, 10617 Taiwan, ROC; 3 Institute of Chemistry, Academia Sinica, No. 128, Section 2, Academia Rd, Nangang District, Taipei, 11529 Taiwan, ROC; 4 Sustainable Chemical Science and Technology, Taiwan International Graduate Program, Academia Sinica, No. 128, Sec. 2, Academia Rd, Nangang District, Taipei, 11529 Taiwan, ROC; 5 Chemistry Department, Faculty of Mathematics and Natural Sciences, Universitas Sebelas Maret, Jl Ir. Sutami 36A, Surakarta, 57126 Indonesia; 6 Department of Chemistry and Biomolecular Science, Faculty of Engineering, Gifu University, Gifu, 501-1193 Japan; 7 Division of Inorganic and Physical Chemistry, Research Center for Nanosciences and Nanotechnology, Center for Catalysis and Reaction Engineering, Institut Teknologi Bandung, Jl. Ganesha No. 10, Bandung, 40132 Indonesia; 8 Faculty of Pharmacy, Universitas Gadjah Mada, Sekip Utara, Yogyakarta, 55281 Indonesia

**Keywords:** Chemistry, Materials science, Nanoscience and technology

## Abstract

Controlling the premature release of hydrophobic drugs like quercetin over physiological conditions remains a challenge motivating the development of smart and responsive drug carriers in recent years. This present work reported a surface modification of mesoporous silica nanoparticles (MSN) by a functional compound having both amines (as a positively charged group) and carboxylic (negatively charged group), namely 4-((2-aminoethyl)amino)-4-oxobut-2-enoic acid (AmEA) prepared via simple mechanochemistry approach. The impact of MSN surface modification on physical, textural, and morphological features was evaluated by TGA, N_2_ adsorption–desorption, PSA-zeta, SEM, and TEM. The BET surface area of AmEA-modified MSN (MSN-AmEA) was found to be 858.41 m^2^ g^−1^ with a pore size of 2.69 nm which could accommodate a high concentration of quercetin 118% higher than MSN. In addition, the colloidal stability of MSN-AmEA was greatly improved as indicated by high zeta potential especially at pH 4 compared to MSN. In contrast to MSN, MSN-AmEA has better in controlling quercetin release triggered by pH, thanks to the presence of the functional groups that have a pose-sensitive interaction hence it may fully control the quercetin release, as elaborated by the DFT study. Therefore, the controlled release of quercetin over MSN-AmEA verified its capability of acting as a smart drug delivery system.

## Introduction

Quercetin (Que), 3,3′,4′,5,7‐pentahydroxyflavone (C_15_H_10_O_7_), is one of the potential dietary compounds existing as a natural polyphenolic flavonoid that can be found in various vegetables, fruits, plant-derived food, and beverages^1,2^. This compound has a wide spectrum of biological activities, especially anticancer activity toward various high-risk cancers such as breast, colon, pancreatic, liver, lung, prostate, bladder, gastric, bone, blood, brain, cervical, eye, and so forth^3,4^. Owing to its dose-dependence effect, this compound serves antioxidant activity at low concentrations but turns to elicit chemotherapeutics effects at high concentrations due to its pro-oxidant functions^5^. At a certain concentration, quercetin could reduce proliferation, induce apoptosis, and inhibit the mitotic process by several pathways such as PI3K/Akt, MAPK, or even through binding in PDK3^6,7^. Unfortunately, this compound has low water solubility hence reducing its bioavailability. Therefore, previous studies have developed smart drug carrier-based polysaccharides^8–10^, liposomal cargos^11,12^, carbon-based particles^13^, and inorganic nanoparticles^14–16^, to address the issue. The role of the drug carrier is to ensure the quercetin reaches the targeted cells hence improving the treatment efficacy. Moreover, the cargo should capable to store high concentrations of quercetin molecules which thereafter boost its chemotherapeutics effect. Among the mentioned drug carriers, mesoporous silica nanoparticles are suitable for entrapping high concentrations of drugs given an advantage of their porous and frameworks nature.

Mesoporous silica nanoparticles (MSN) has received considerable attention as potential drug carrier owing to their properties, such as tunable particle and pore size, well-defined and rigid porous structure and framework, high surface area to volume ratio, enriched by hydroxyl groups for further modification, and biocompatible^17^. This nanomaterial, however, lacks in controlling drug release which inflicts inefficiency and ineffective disease treatments^18^. Therefore, further modification is critically important to overcome the shortcoming of this material. Xu et al.^14^ developed poly(2-(diethylamino)ethyl methacrylate) modified silica nanoparticles via photo-induced atom transfer radical polymerization, exhibiting a pH-controlled drug release indicated by a high drug concentration released at pH 5.5 than pH 7.4. In another investigation, Chen et al.^19^ employed polydopamine-coated hollow mesoporous silica (HMS) nanoparticles to achieve controllable drug release by pH stimuli. The controlled release mechanism was based on the self-degradation of polydopamine under acidic conditions resulting in more than 40% drug released at pH 6.5, while it is only 25.63% at pH 7.4. Other researchers also employed functional amino-based polymers, such as polyethyleneimine^20^, polypeptides^21^, chitosan^22^, etc., to create smart features for drug delivery systems. Despite their controlled-release performance, surface modification of MSN by polymeric substances inherently induces a pore-blocking effect which impacts declining textural properties, enlargement particle size, and also having toxicity issues. Moreover, the complexity of the multi-step synthesis procedure, including, polymeric synthesis, surface activation of nanoparticles, polymerization or polymeric conjugation, and purification, is also one of the obstacles which in some cases involve uncertain toxic substances and loosing of drugs during preparation. Therefore, rapid and facile surface functionalization of MSN still becomes a challenge in this research field.

The involvement of organosilane in the surface engineering of MSN could compromise the premature release issue of the MSN drug carrier platform. The organic moieties on the surface of organosilane-modified MSN act as the gatekeeper in controlling the release of drug molecules via light^23^, pH^24^, ultrasound^25^, thermal^26^, redox^27^, and other stimuli, which successively improve therapeutic efficacy. In addition, this type of nano-carriers offers biodegradable features given by organic functional groups under certain biological conditions^26,28^. Previously, we have prepared amino-based organosilane as surface motifs of core–shell Fe_3_O_4_@MSN nanoparticles which controlled the release behavior of curcumin triggered by pH^29^. The presence of such amine motifs on nanoparticle surfaces suppresses the drug release at artificial physiological conditions (~ 10%), while more drug escapes within nanoparticles under an artificial cancer environment (49.12%). Alswieleh et al. investigated the drug loading and release abilities of three different functional groups of organosilane (such as amines, thiol, and sulfonate) anchored along internal MSN pores. They found that the MSN-containing sulfonate group has higher drug loading efficiency, while the MSN modified by either amines or sulfonate groups has a controlled release feature over the pH stimuli^30^. In another study, multifunctional groups, consisting of tertiary amines and carboxylic groups, were successfully introduced on the MSN surfaces via a multilayer synthesis method exhibiting a multiple-responsive behavior (enzymatic, glutathione, and pH-sensitives)^31^. The presence of carboxylic and amines on the surface of MSN mimicked a zwitterion-like structure that switched from negatively charged to positively charged or vice versa upon changing the environment. This leads to the release of drugs specifically targeted in the cancer environment. However, this strategy requires several steps in the synthesis of nanoparticles which have an impact on enlarging the size of the nanoparticles and generating more chemical waste. Therefore, our approach designed a simple, rapid, and facile protocol to engineer the surface of MSN involving thiol-ene click reaction to provide both amines and carboxylic groups not only on MSN surfaces but also on the pore channels.

This present work demonstrates the viability of MSN surface engineering by involving the thiol-ene click chemistry approach to introduce amines and carboxylic motifs in controlling the quercetin release behavior. The molecular motif, we called 4-((2-aminoethyl)amino)-4-oxobut-2-enoic acid (AmEA), has been simply prepared by mechanochemistry and further docked into the thiol-enriched MSN surface. As we hypothesized, the modified MSN (MSN-AmEA) had higher effective drug loading and was better in controlling drug release compared to the pristine MSN proclaiming a positive impact on our approach. This study also discusses the kinetic release of quercetin to portray its release mechanism from nanoparticles which are also supported by the DFT study.

## Methods

### Materials

Tetraethyl orthosilicate (TEOS 98%), mercaptopropyl trimethoxysilane (MPtMS), ammonium hydroxide (NH_4_OH), azobisisobutyronitrile (AIBN 12 wt% in acetone), and dimethylformamide (DMF) were purchased from Sigma-Aldrich. Maleic anhydride, ethylenediamine, hexadecyltrimethylammonium bromide (HtAB), ethanol, and methanol were commercially available from Merck. Quercetin dehydrate (C_15_H_10_O_7_∙2H_2_O) was purchased from Solarbio. Ultrapure water was acquired from Integrated Laboratory, Universitas Sebelas Maret. The PBS buffer was prepared by dissolving NaCl (8 g, Merck), KCl (0.2 g, Merck), Na_2_HPO_4_ (1.44 g, Merck), and KH_2_PO_4_ (0.24 g, Merck) in 1000 mL of deionized water. The pH was adjusted by adding HCl (Mallinckrodt) and NaOH (Merck).

### Synthesis of 4-((2-aminoethyl)amino)-4-oxobut-2-enoic acid (AmEA)

Synthesis of AmEA compound was conducted by modifying procedure reported by previous work^32,33^. Briefly, maleic anhydride (1.961 g, 0.019 mol) was crushed into fine powder. Subsequently, ethylenediamine (1.201 g, 0.019 mol) was dropped wisely and grounded thoroughly using mortal and pastel for 30 min. A sticky wet texture was obtained and aged for 24 h. Afterward, the product was washed with a mixture solution of ultrapure water and ethanol (1:1 v/v), slowly evaporated at mild temperature to yield a viscous solution then cooled down at room temperature. The yellowish flower-like crystals were acquired, washed with acetonitrile, and dried in vacuum. The compound was 4-((2-aminoethyl)amino)-4-oxobut-2-enoic acid (denoted as AmEA).

### Synthesis and functionalization of MSN

Mesoporous silica nanoparticles were synthesized via the sol–gel method. Typically, HtAB (0.78 g, 2.14 mmol) was dissolved in a mixture solution of 21.6 mL deionized water and 3.4 mL ethanol, followed by the addition of TEA (53 µL, 0.0004 mmol). The mixture was stirred for 1 h at 60 °C in the silicon oil bath. Afterward, 2 mL of TEOS was slowly added and kept under mild stirring conditions for 2 h. The MSN was collected by centrifugation and washed several times using deionized water. The HtAB template was removed by the reflux method followed by our previous work^18^.

The functionalization of MSN was performed through two steps. The first step was silylation to introduce the thiol groups on the MSN surfaces. The second step was a thiol-ene ‘click’ reaction. Briefly, 0.5 g of MSN was dispersed in 25 mL of toluene through sonication for 10 min. Then, 150 µL of MPtMS was added and was sonically treated for 20 min. The reaction was continued by refluxing the mixture for 16 h. The silylated MSN (s-MSN) were collected by centrifugation, washed with fresh toluene, ethanol, and deionized water sequentially, and followed by drying through lyophilization. The dried s-MSN were dispersed in 20 mL deionized water followed by the addition of 705 µL AIBN. Afterward, 0.08 g AmEA was added to the suspension. The reaction was maintained under a nitrogen atmosphere under UV 365 nm irradiation for 6 h. afterward, the product was washed with deionized water and dried using a freeze-drying machine. The functionalized product was denoted as MSN-AmEA.

### Loading procedure of quercetin onto nanoparticles

The quercetin was loaded onto both MSN and MSN-AmEA by a slow evaporation method. Quercetin (10 mg) was dissolved in 10 mL of ethanol. Then, nanoparticles with a ratio of 3:1 w/w were added to quercetin solution and dispersed by sonication. The mixture was mildly stirred until the ethanol was completely evaporated. The nanoparticles containing quercetin (Que@MSN or Que@MSN-AmEA) were washed with deionized water and dried in the freeze-drying machine. The effectiveness of adsorption was evaluated by dispersing 5 mg of nanoparticles containing quercetin into 5 mL of ethanol. The supernatant was collected and the absorbance was measured using a UV–Vis spectrophotometer at 372 nm. The adsorption effectiveness (AE) was defined as the effective amount of drugs loaded onto nanoparticles and was calculated following Eq. (1). Afterward, the adsorption capacity of each nanoparticle can be calculated following Eq. (2)

1 $$AE \left(\%\right)=\frac{(C-{C}_{e})}{C}\times 100\%,$$

2 $$Qe \left(\mathrm{mg} {\mathrm{g}}^{-1}\right)=AE\times \frac{{m}_{Q}}{{m}_{D}},$$

where, C and C_e_ are the initial and unloaded (contained in the supernatant after washing) concentrations of quercetin, respectively. m_Q_ (mg) and m_D_ (g) are the weight of quercetin and drug carrier.

### Kinetic release study of quercetin

The release study was performed in two pH conditions of PBS buffer, namely pH 7.4 and 4.0 representing physiological and cancer cells microenvironments. About 5 mg of each nanoparticle containing quercetin was immersed in 10 mL of PBS solution and shaken at 120 rpm. After certain time (1, 2, 4, 8, 16, 24 h and afterward every 12 h of time points), about 1 mL of supernatant was collected for UV–Vis measurement. Then, 1 mL of fresh PBS solution was added into the sample to replace the taken solution. The quercetin released from nanoparticles was quantified by measuring the absorbance of the solution at 377 nm. Then, the concentration of quercetin was determined by calibration curve (Supplementary Fig. S1). The release of quercetin from nanoparticles was calculated using Eq. (3).

3 $$Release \left(\mathrm{\%}\right)= \frac{\mathrm{C }\times V}{{\mathrm{Q}}_{e}\times m}\times 100\mathrm{\%},$$

where C is the amount of quercetin released to the system (mg L^−1^); V is the volume of the system (L); Qe is quercetin adsorption capacity of nanoparticles (mg g^−1^), and m is the mass of nanoparticles containing quercetin (g).

The kinetic release of both MSN and MSN-AmEA was studied by following zero-order, first-order, Ritger-Peppas and Higuchi kinetic release models. The experiment data were fitted using a respective nonlinear equation according to Eq. (4) (zero-order), Eq. (5) (first-order), Eq. (6) (Ritger-Peppas), and Eq. (7) (Higuchi)^34^.

4 $$F={M}_{t}/{M}_{inf}={k}_{0}t,$$

5 $$F={M}_{t}/{M}_{inf} =100\times \left(1-{e}^{-{k}_{1}t}\right),$$

6 $$F={M}_{t}/ {M}_{inf}={k}_{RP}{t}^{n},$$

7 $$F={M}_{t}/ {M}_{inf} ={k}_{h}{t}^{0.5}.$$

*M*_*t*_ and *M*_*inf*_ are cumulative drug releases at time t and equilibrium, respectively. *F* is the fraction of drug release at time t. *n* is the diffusion exponent. *k*_*0*_, *k*_*1*_, *k*_*RP*_, and *k*_*H*_ are the kinetic release rate constant of zero-order, first-order, Ritger–Peppas, and Higuchi models.

### Characterization of materials

Functional groups of the sample were analyzed by FTIR (Fourier Transform Infra-red) IR Prestige-21 Shimadzu. The sample was recorded using a KBr pellet and scanned in the range 4000–400 cm^−1^, with a resolution of 4 cm^−1^.

The ^1^H-NMR of AmEA was recorded by Nuclear Magnetic Resonance (NMR) model Bruker AV300. The AmEA compound was diluted in 0.5 mL of D_2_O for analysis.

Morphology of the sample was observed using Scanning Electron Microscopy (SEM) and Transmission Electron Microscopy (TEM). The nanoparticles were sticked to carbon tape and coated by ruthenium using spin coating machine prior SEM measurement. The SEM images were taken by Field Emission SEM (FESEM) model Hitachi S-4800. For TEM measurement, the samples (around 5 mg in 1 mL ethanol) were pretreated in ultrasonic bath. About 10 μL was dropped to Cu grid and dried at room temperature. The images were captured using TEM model JEOL-JEM 2100 with accelerating voltage of 200 kV.

The N_2_ adsorption–desorption isotherm was studied by Surface Area Analyzer (SAA) Quantachrom Nova e1600 with a degassing temperature of 180 °C. Before measurement, the nanoparticles were dried in oven overnight at 100 °C. About 20 mg of nanoparticles was used for this measurement and degassed using N_2_ gasses for 4 h.

The thermal properties of nanoparticles were analyzed by thermogravimetric analyzer (TGA) and differential scanning calorimeter (DSC) with model STA Lineises PT-1600. The TGA data was taken in range temperature of 20–600 °C with the heating speed of 10 °C per minutes.

The hydrodynamic properties of nanoparticles were characterized using particle size analyzer (PSA) model Malvern Zetasizer. The nanoparticles were suspended in PBS buffer (concentration of 50 mM) before measurement.

### Molecular electrostatic potential and DFT study

Molecular electrostatic potential (MEP) of quercetin and MSN-AmEA surface moieties was obtained through the DFT B3LYP approach with the exchange–correlation function and basis set of 6-31g**. The density radius was set at level r = 0.001^35,36^. Visualization of optimized geometry and MEP was done by Chimera 1.12^37^. The interaction of MSN-AmEA surface moieties and quercetin were calculated using Gaussian 09, in which the quercetin structure was acquired from the material bank (CID 5280343) and was optimized using DFT method with basis set 6-31g**. The MSN-AmEA surface moieties and quercetin were approached at a distance of 2–2.5 Å from the active groups of each compound. The Si–O groups on the MSN-AmEA were set to be rigid, meanwhile, other groups were flexible. The calculation was performed using the DFT method with basis set 6-311G** and the position of each compound was varied.

### Cell uptake and viability study

The neuroblastoma cells were cultured in DMEM medium (containing 10% FBS) in 12-well plate for 24 h in 5% CO_2_, 95% air at 37 °C. Afterward, the FITC labelled MSN and MSN-AmEA (in DMEM medium) was added to the cultured cell to achieve final concentration of 0, 1, 10, and 100 µg mL^−1^. The nanoparticles treated cells were incubated again for 4 h and then fixed using 4% paraformaldehyde. After incubation for 20 min, the cell was washed with PBS and then treated with 0.2% triton-X for 10 min. Then, DAPI (5 µg mL^−1^) was added to the fixing cell to label the nucleus. The cell containing nanoparticles was then analyzed using fluorescence microscope at DAPI and FITC channels.

The viability test was conducted using Alamar blue protocol. Briefly, the neuroblastoma cells were cultured in 96-well plate for 24 h. Afterward, the MSN, MSN-AmEA, and Que@MSN-AmEA was added to the cultured cell and incubated again for another 24 h. The alamar blue (10 µL) was added into the 96-well plate containing nanoparticles treated cell (90 µL), and treated for 4 h. Then, the absorbance (570 nm) of solution was measured by using ELISA-Reader. The cell viability of each nanoparticle was calculated according to Eq. (8).

8 $$\% Cell \; viability= {A}_{570}treated \; cells / {A}_{570}Control \times 100\%.$$

## Results and discussion

### Structural analysis of AmEA functional compound

**Figure 1:**
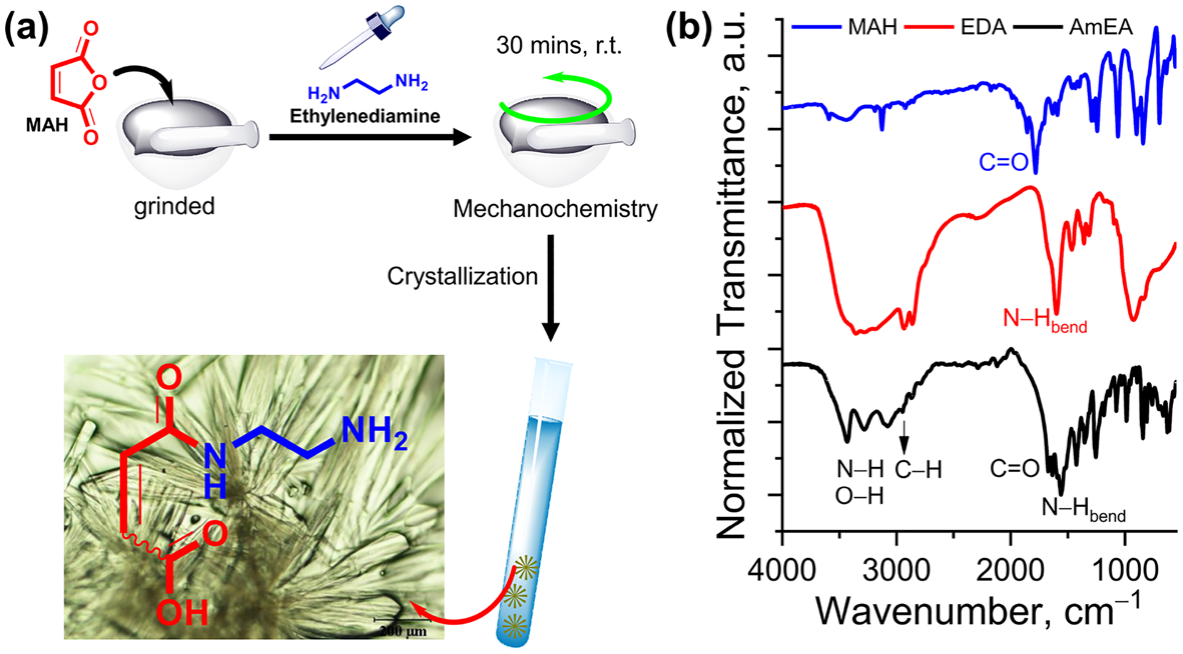
(**a**) Scheme synthesis and photograph of AmEA compound (created by using ChemDraw Professional version 20.0.0.41, https://www.cambridgesoft.com/Ensemble_for_Chemistry/ChemOffice/ChemOfficeProfessional). (**b**) FTIR spectra of MAH, EDA and AmEA compounds.

**Figure 2:**
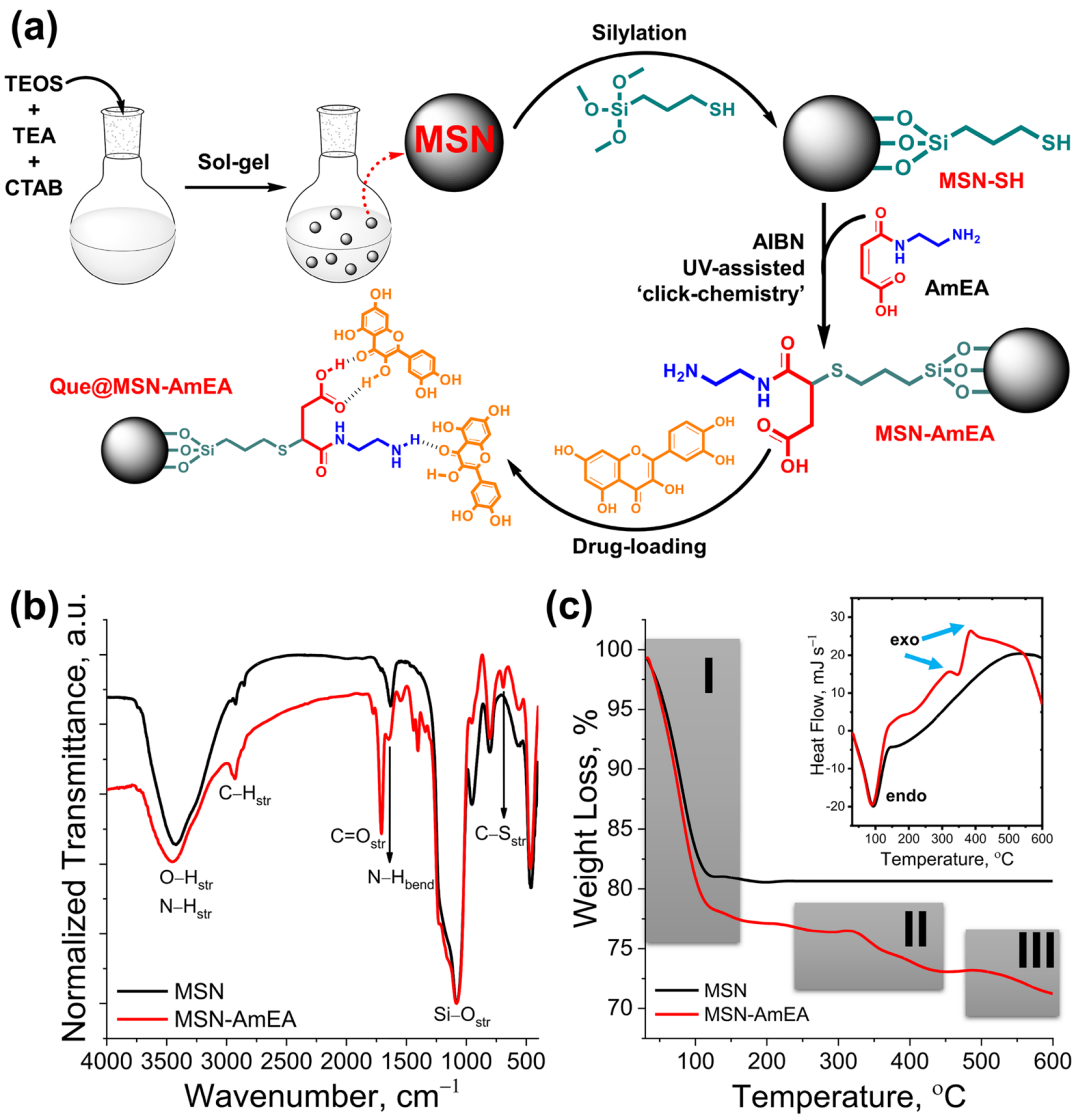
(**a**) Illustration synthesis of MSN-AmEA via thiol-ene ‘click chemistry’ consisted of MSN synthesis through the sol–gel method, silylation of MSN to introduce thiol group and chemically conjugated AmEA by the thiol-ene reaction. The scheme also presents the plausible interaction between quercetin and MSN-AmEA through hydrogen bond interaction (created by using ChemDraw Professional version 20.0.0.41, https://www.cambridgesoft.com/Ensemble_for_Chemistry/ChemOffice/ChemOfficeProfessional). (**b**) FTIR spectra of MSN and MSN-AmEA indicate a successfulness of AmEA conjugation onto MSN surfaces. (**c**) TGA and DSC (inserted box) curves of MSN-AmEA exhibit an additional two-stage degradation undergoing exothermic thermal degradation.

The AmEA compound was synthesized through a simple amidation reaction facilitated by the mechanochemistry approach. Figure 1(i) illustrates the chemical synthesis of the AmEA compound derived from maleic anhydride and ethylenediamine. The addition of ethylenediamine should be gently and slowly added due to the exothermic reaction. In this study, we used 1:1 molar ratio to ensure only AmEA products formed without any side dimeric product. The AmEA has a light green flower-like crystal as observed under optical microscopy as shown in Fig. 1a. The chemical structure of AmEA was confirmed by FTIR and ^1^H-NMR characterizations as revealed in Fig. 1b and Supplementary Fig. S2, respectively. Structurally, the AmEA compound has hydroxyl, amines, carboxyl and vinyl groups. Those functional groups can be identified by using FTIR analysis. For example, peak at 1672 cm^−1^ is associated with the C=O stretching vibration. Meanwhile, bands at 3431 and 3280 cm^−1^ correspond to the symmetric and asymmetric N−H stretching vibration of primary amine, while the secondary amine can be found at 3074 cm^−1^ which overlaps with the O−H stretching vibration. The band at 1591 cm^−1^ is the characteristic peak for stretching vibration of the C=C bond. We also analyzed the ^1^H-NMR of this compound as shown in Supplementary Fig. S2. The data indicates peaks at chemical shift of 6.29 ppm (d, *J* = 12.2 Hz, 1H) and 6.04 (d, *J* = 12.1 Hz, 1H) revealing a proton signal of typical Z-isomer at C=C bond. Signals also found at chemical shift of 3.53 ppm (t, 2H) and 3.16 ppm (t, 2H) which correspond to the proton signals at − CH_2_ near amide bond and − CH_2_ close to primary amines. Based on the ^1^H-NMR data, the AmEA compound has a mixture isomer (E and Z form). However, we did not perform a purification due to both of the isomer will have a same product after thiol-ene reaction as illustrated in Fig. 2a. Owing to its functional groups, the AmEA compound has an advantage in enriching MSN surface moieties to improve drug storage or release performance. The vinyl group will facilitate a chemical conjugation to the MSN surfaces via a thiol-ene click reaction. Meanwhile, the amine and carboxylate groups play a critical role in assisting chemical interaction with quercetin which affects the loading and release performance of the nanocarrier^38^.

### Functionalization of MSN via ‘thiol-ene’ click chemistry

The plausible chemical reaction involved during surface functionalization via thiol-ene reaction was proposed in Fig. 2a. The MSN was obtained by the simple sol–gel method and was further silylated using a thiol-end group organosilane compound. These thiol groups allow the MSN to chemically react with the vinyl group of AmEA via thiol-ene ‘click’ chemistry. This approach was one of the powerful and versatile tools for non-catalytic heteroatom bond formation yielding high atom economy, facile synthetic pathway, and relatively high reaction rate, so-called “green” protocol^39^. Under UV irradiation and further thermal lysis approaches, the thiol group was promoted into thiyl radical which was assisted by AIBN radical initiator^40,41^. This form makes AmEA easily conjugate onto MSN surfaces by opening the C=C bond through sharing electron (forming radical) and further covalently bonded with thiyl radical which resulted in the C−S bond. Finally, another side of the C radical will be terminated by a proton radical to form a structure as exhibited in Fig. 2a. The rich functional groups over MSN-AmEA possibly enhance the capability of these materials to interact with quercetin hence significantly improving the loading amount. Hereafter, we investigated the chemical and physical properties of both MSN and MSN-AmEA.

The preliminary study by FTIR measurement designated that thiol-ene click reaction was successfully applied to the surface functionalization of MSN. Figure 2b depicts FTIR spectra of MSN and MSN-AmEA demonstrating a significant change that was visually observed through both of the spectra. A broad peak at around 3420 cm^−1^ on MSN FTIR spectra is the hydroxyl group typical absorbance band. This peak was also found in the MSN-AmEA FTIR spectra with a slightly shifting to around 3445 cm^−1^. Additionally, the peak is broader which is probably due to the contribution of the N−H stretching vibrations originating from the AmEA structure. Moreover, new peaks at around 1706 cm^−1^ and 1642 cm^−1^ are also found on the MSN-AmEA spectra, associated with the C=O stretching and N−H bending vibrations, respectively. The C=C and S−H characteristic vibrations were not found in the FTIR spectra of MSN-AmEA indicating that all vinyl groups were completely reacted with thiol groups forming C−S bond. This bond can be easily distinguished by the FTIR technique, in which a new weak peak at 702 cm^−1^ is a characteristic peak of C−S stretching vibration^42^. Therefore, this preliminary data supported the plausible structure of MSN-AmEA which was shown in Fig. 2a.

Figure 2c depicts the TGA and DSC profiles of both MSN and MSN-AmEA. The MSN has only one degradation curve initiated at the beginning of measurement up to around 110 °C. This degradation profile was the loss of water molecules entrapped either on the surface or inside the pore channels of MSN. About 18% of water molecules loss was quantified in the MSN as observed by TGA which undergoes endothermic reaction as pointed out by DSC data (inserted box of Fig. 2c). The porous silica-based material is often found to have good thermal stability indicated by no degradation occurring above 200 °C up to 600 °C, which was also found by Oboudatian and Safaei-Ghomi^43^. The MSN-AmEA, on contrary, has three degradation profiles, clearly revealed in Fig. 2c. The first degradation is the loss of 23% of water molecules. The mass loss of water molecules in MSN-AmEA was higher than MSN which could be associated with the presence of AmEA polar functional groups such as amines, and carboxylic groups. These moieties facilitate hydrogen bonding to water molecules thus improving the hydrophilicity of nanoparticles^44^. Consequently, more water was entrapped within the surface or internal pores. The second and third degradation that occurred above 340 °C were associated with the degradation of AmEA molecules that chemically conjugated to nanoparticles surfaces and followed by thermal decomposition of the whole MPtMS chain in the last stage^45^. Both of them were exothermic reactions as indicated by the DSC curve. The presence of these additional two degradation profiles suggests that either silylation or chemically conjugation of AmEA on the MSN nanostructure was successfully achieved.

### Morphological and textural properties of nanoparticles

**Figure 3:**
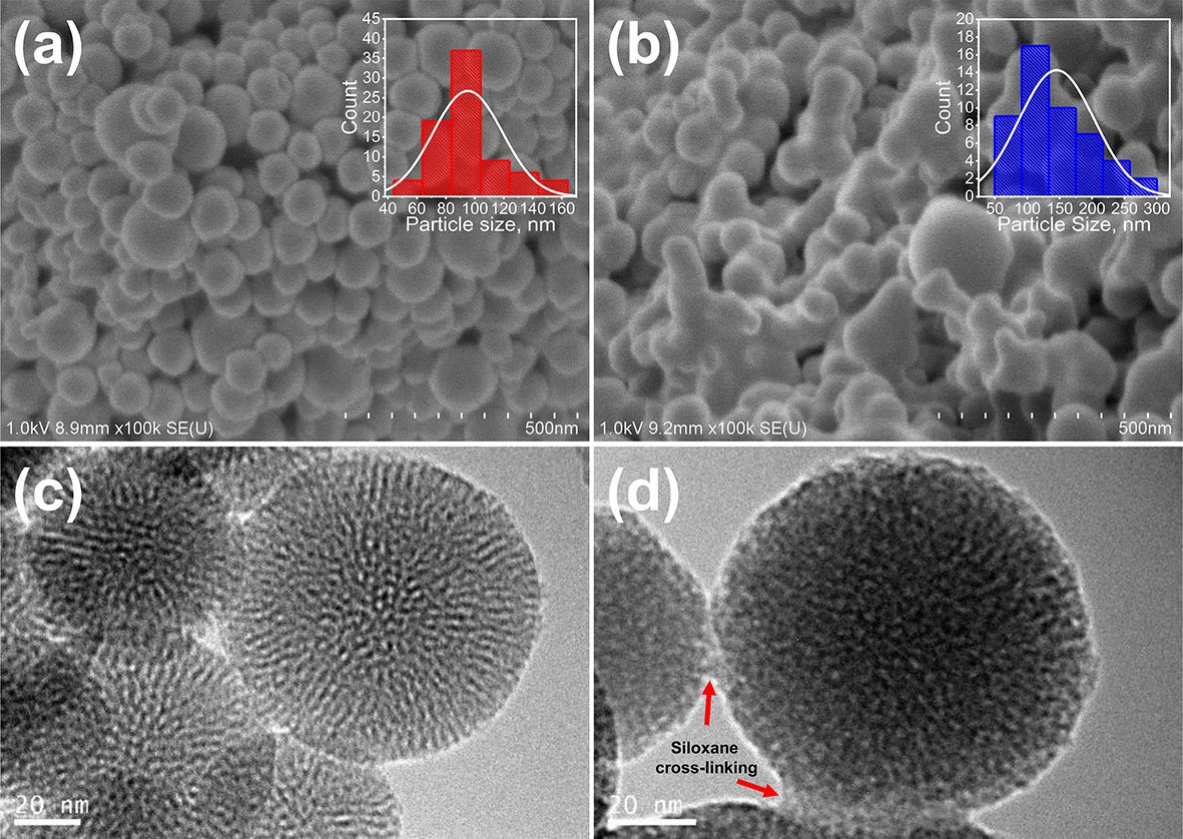
SEM images of (**a**) MSN and (**b**) MSN-AmEA, the inserted box with red and blue graph color indicate the particle size distribution of nanoparticles. TEM images of (**c**) MSN and (**b**) MSN-AmEA. The siloxane network of organosilane compounds linked to the spherical MSN is observed by TEM.

Morphology of MSN and MSN-AmEA was observed by SEM and TEM and the result can be seen in Fig. 3. The MSN has a uniform spherical nanostructure with an average particle size of 95.17 ± 23.89 nm as depicted in Fig. 3a. Deeply, the porous structure of MSN was orderly and well-divinely formed in which the cylindrical channels were observed by TEM measurement as confirmed in Fig. 3c. The impact of surface modification on the morphological structure of MSN can be experientially observed in Fig. 3b,d. It was found that the nanoparticles seem coated and collated which led to an increase in particle size to 145.41 ± 58.38 nm. In addition, the porous structure of MSN-AmEA looks clogged, but the cylindrical form is still distinguished. This suggests that the silylation occurred not only on the surface of nanoparticles but also in the pore channels. The silylation of MSN leads to the formation of a siloxane network by polycondensation reaction causing the occurrence of interconnecting particles. In fact, controlling this phenomenon are quite difficult, but in some cases, it can be done by limiting the concentration of organosilane compound. However, it will cause a defect of the surface functional group, in which some sites do not have active functional groups and limit their interaction with the drug molecules hence reducing the loading efficiency. Therefore, this technique remains a challenge for future work.

**Figure 4:**
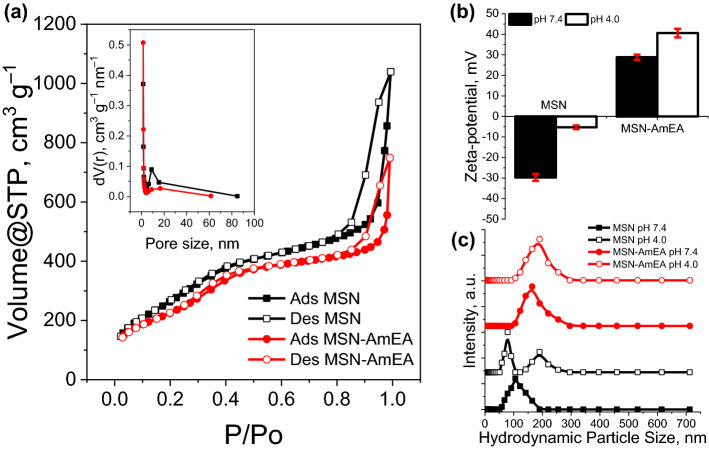
(**a**) N_2_ adsorption–desorption isotherm (inserted box represented the pore size distribution), (**b**) zeta-potential, and (**c**) hydrodynamic particle size of MSN and MSN-AmEA.

The textural properties of both MSN and MSN-AmEA were evaluated by N_2_ adsorption–desorption isotherm and dynamic light scattering methods. Figure 4a depicts the hysteresis loop and pore size distribution of both MSN and MSN-AmEA. Generally, both nanoparticles are classified as type IV with hysteresis close to H1 reflecting mesoporous materials with cylindrical pore structure. This data was in good agreement with the TEM measurement showing the cylindrical pore channels of the nanoparticles. The BET surface area of MSN was found to be 1038.53 m^2^ g^−1^ with pore volume and average pore size of 1.61 cm^3^ g^−1^ and 3.09 nm, respectively. The surface modification of nanoparticles, on the other hand, decreased the BET surface area, pore volume, and average pore size as presented in Table 1. The previous TEM analysis revealed that the surface of MSN-AmEA was covered by polysiloxane network. This induced the pore-blocking effect making the N_2_ difficult to access its pore channels^18,29^. Moreover, the increase in particle size also contributed to this issue. The organosilane undergoes polycondensation forming Si–O–Si networks and could connect the nanoparticles which leads to enlargement of the particle size. By comparing the size of both nanoparticles, MSN-AmEA has a larger particle size than MSN hence resulting in a smaller BET surface area. Even though, the adsorption–desorption isotherm hysteresis pattern of MSN-AmEA is similar to MSN indicating its cylindrical porous characteristic.

**Table 1:** Textural properties of MSN and MSN-AmEA, including BET and Langmuir surface area, pore volume, pore diameter, average particle size (L) measured by SEM, hydrodynamic particle size by DLS method, zeta (ζ) potential, and polydispersity index (PDI).

Materials	A_BET_ (m^2^ g^−1^)	A_Langmuir_ (m^2^ g^−1^)	V_pore_ (cm^3^ g^−1^)	d_pore_ (nm)	L^a^ (nm)	DLS^b^ (nm)	ζ-pot^b^ (mV)	PDI^b^
MSN	1038.53	1724.98	1.61	3.09	95.17 ± 23.89	107.99 ± 1.18	− 29.7 ± 1.7	0.23 ± 0.014
MSN-AmEA	858.41	1431.08	1.16	2.69	145.41 ± 58.38	167.33 ± 0.94	28.3 ± 1.3	0.45 ± 0.022

^a^Average particle size (L) was calculated using SEM images.

^b^Measured under PBS solution pH 7.4.

To evaluate the surface charges and hydrodynamic particle size of nanoparticles under PBS buffer at pH 7.4 and 4.0, the DLS measurement was performed in this study as indicated in Fig. 4b,c. These measurements are very important before either in-vitro or in-vivo investigation to justify whether the nanoparticles can be decently applied. The MSN has a negative zeta potential value, denoting negative surface charges, both in pH 7.4 and 4.0 PBS conditions. The zeta potential of MSN is − 29.7 mV at pH 7.4. However, the value was close to the isoelectric point (− 5.3 mV) at pH 4.0. This infers good suspension stability of MSN at pH 7.4 but tends to coalesce at acidic conditions owing to electrical double layer suppression by changing the charge equilibrium. In many cases, MSN indeed has a negatively surface charge due to the Si−(OH) groups. This condition is unfortunately not preferable for biomedical purposes, especially for drug delivery systems. The silanol groups have unfavorably interacted with membrane lipid and plasma proteins, hence could destroy their structure and causing toxicity^46^. Therefore, this surface modification, by engineering the MSN surface functional group, is subjected to substitute the surface charges and improve the biocompatibility of the MSN. Introducing AmEA on the surfaces of the nanoparticles exchanged the net surface charge to be more positive at both pH 7.4 and 4.0 conditions. The zeta-potential of MSN-AmEA at pH 7.4 is + 28.8 mV and increases to + 40.6 mV at pH 4.0, thereby highly stable in the colloidal system. We are expectedly satisfied with these results, in which the positive surface charge of nanoparticles offers better cellular uptake than negatively surface change nanoparticles by internalization through the cell membrane via positive–negative electrostatic interaction^47^. Therefore, we believe that the MSN-AmEA can be compromised with biocompatibility and toxicity issues.

The hydrodynamic particle size distribution of MSN and MSN-AmEA was depicted in Fig. 4c. In this study, we did not optimize the dispersant media, but directly use PBS solution to understand the hydrodynamic size of our materials. The PBS buffer was prepared in two pH conditions mimicking the physiological condition (pH 7.4) and acidic environment (pH 4.0). Under physiological conditions, both hydrodynamic particle sizes of MSN and MSN-AmEA are close to the SEM measurement, which is 107.99 nm (PDI = 0.23) and 167.33 nm (PDI = 0.45), respectively. However, the polydispersity hydrodynamic particle size distribution of MSN was indicated at pH 4.0 which was signified by the presence of two peaks with the PDI value of 0.97. In this state, we found the MSN precipitate after the DLS measurement expressing its colloidal instability as proven by low zeta-potential value (close to zero). On the contrary, this phenomenon was not found in MSN-AmEA in which the hydrodynamic particle size at both pH conditions was almost the same. At pH 4.0, the MSN-AmEA has 178.49 nm of hydrodynamic particle size with a PDI of 0.36. There was no precipitate observed at this condition which compromised to zeta-potential value and reflected highly colloidal stability.

### Adsorption and release of quercetin over nanoparticles

**Figure 5:**
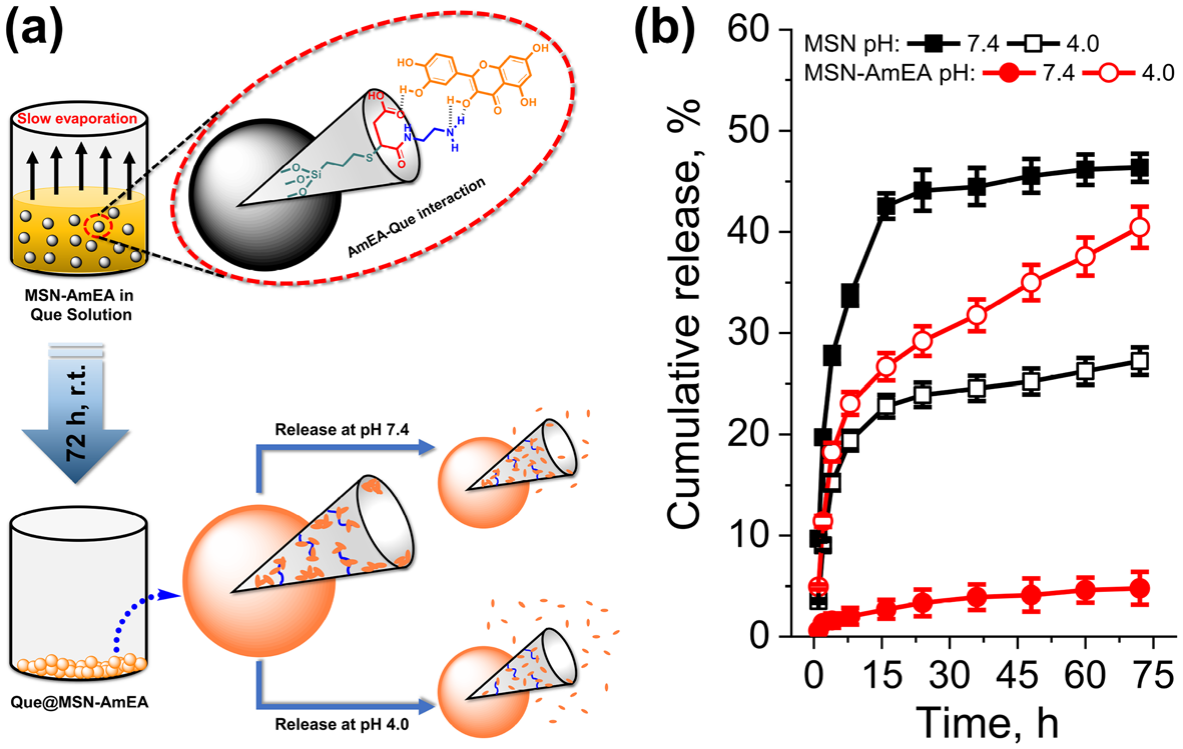
(**a**) The scheme illustrated the loading process of Que onto MSN-AmEA, and how they possibly interacted, also the representation of Que released from nanoparticles at pH 7.4 and 4.0 (created by using ChemDraw Professional version 20.0.0.41, https://www.cambridgesoft.com/Ensemble_for_Chemistry/ChemOffice/ChemOfficeProfessional). (**b**) The cumulative release profiles of Que@MSN and Que@MSN-AmEA at pH 7.4 and pH 4.0.

The adsorption study was performed via the slow evaporation method to maximize the loading capacity of the drug carriers. The quercetin molecules were forced to adsorb onto drug carriers and expected to diffuse and fill their entire porous structures. It was found the effective adsorption of MSN was about 45.48% (Qe = 136.54 mg g^−1^) and doubled up after being engineered (MSN-AmEA) for about 97.52% (Qe = 297.45 mg g^−1^). The high adsorption of quercetin within MSN-AmEA is due to the presence of either amines and carboxylic groups that facilitate interaction with the drug molecules via hydrogen bond or electrostatic interaction as illustrated in Fig. 5a^10^. We also performed a DFT analysis to support this data, by interacting surface moieties of MSN-AmEA with quercetin molecules. Interestingly, both amines and carboxylic groups provide a good interaction with quercetin molecules which will be further described in detail in the next section. Based on this result, we confidently stated that our approach to engineering the MSN surface is the best way to optimally improve its loading efficiency.

The pH-dependent feature of MSN and MSN-AmEA was studied through in-vitro drug release in the PBS solution mimicking physiological and acidic environment conditions. Two PBS buffer with different pH was prepared, i.e. pH 7.4 and 4.0, to simulate the quercetin drug release behavior. Figure 5b reveals the cumulative release profile of quercetin over time from Que@MSN and Que@MSN-AmEA samples. The bare MSN has a relatively high quercetin cumulative release of 46.35% at pH 7.4 but decreases to 27.22% at pH 4.0. According to the result, the equilibrium release of quercetin from MSN at pH 7.4 was nearly twice as high as in the acidic condition. The release of drugs depends on the surface properties of the carrier. The MSN have negative surface charges at pH 7.4, and their zeta potential is close to IEP at pH 4.0. Repulsion forces between negatively surface charges of MSN and hydroxyl group enriched quercetin induced the burst release of quercetin into the aqueous system.

Surface engineering of MSN controls the release behavior of quercetin over the pH as presented in Fig. 5b. A low concentration of quercetin (below 5%) was accumulatively released at pH 7.4 indicating a delayed release profile. Meanwhile, at pH 4.0, the profile manifested a sustained release showing no plateau observed up to 72 h. The cumulative release of quercetin from MSN-AmEA at pH 4.0 is about 40.45% and incrementally increases as a function of time. This is probably due to the presence of amines and carboxylic groups on the surface of MSN-AmEA controlling the release mechanism of quercetin. According to zeta-potential measurement, the MSN-AmEA has positive charges both at pH 7.4 and 4.0, influencing the electrostatic interaction (attraction or repulsion) with quercetin as a function of pH condition. Quercetin, owing to its phenolic OH groups at positions 3 and 7, undergoes a dissociation providing more negative charges species by the increasing of pH, about − 48.1 ± 1.2 mV at pH 7 as found by Zembyla et al.^48^ The negatively charged quercetin may lead to a strong electrostatic interaction with positively charged MSN-AmEA resulting in a few quercetin molecules released at pH 7.4. However, quercetin molecules will be protonated at the acidic condition which interferes with their interaction. The interaction between protonated quercetin and positively charged MSN-AmEA may cause repulsion forces inducing a sustained release of quercetin over time.

Quercetin as natural flavonoid compound serves as anticancer. A study by Hashemzaei et al., reported a capability of quercetin inducing the apoptosis of cancer cells. They found that the IC_50_ of quercetin inducing toxicity to the MCF-7 cancer cell was about 105.4 ± 5.2 µM for the treatment time of 24 h^49^. Our study revealed that the cumulative release of quercetin from MSN-AmEA was about 29% at 24 h which equal to 142.7 µM. Therefore, this material has a beneficial effect to be used as drug carrier for treatment of cancer cells.

### The kinetic release study

**Figure 6:**
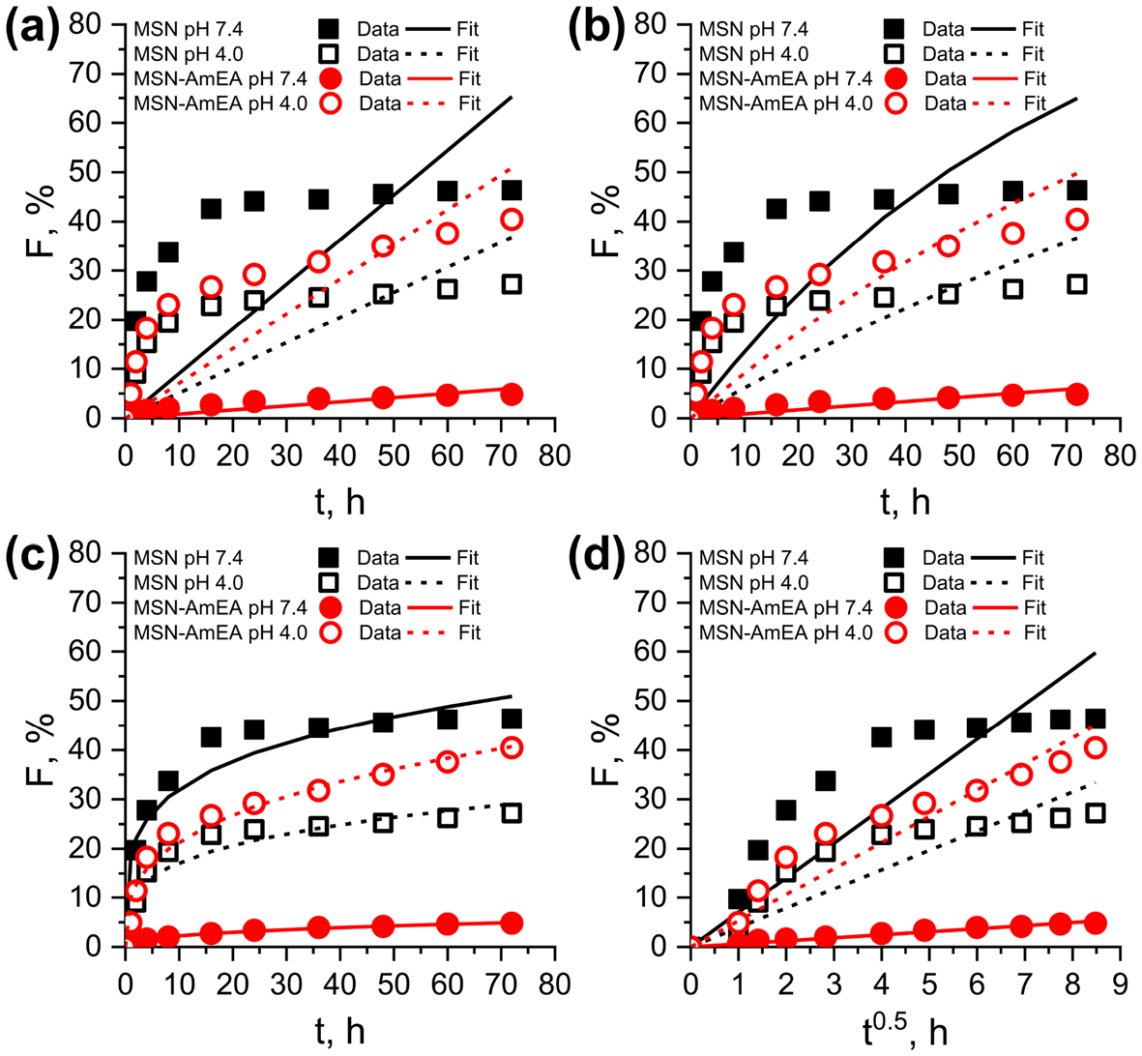
Non-linear fitting graph of quercetin release data from nanoparticles at pH 7.4 and 4.0 with the (**a**) Zero-order, (**b**) First-order, (**c**) Ritger–Peppas, and (**d**) Higuchi kinetic release models.

To better understand the release mechanism of quercetin from the nanocarriers, the kinetic release study was conducted. We applied four kinetic release models, i.e. Zero-order, First-order, Ritger–Peppas and Higuchi models to study the release mechanism of quercetin from nanoparticles. Figure 6a–d shows the fitting quercetin release data at pH 7.4 and pH 4.0 with the kinetic release models^18^. The results, including kinetic rate constant *k*, diffusion exponent *n*, and correlation coefficient *R*^2^ were summarized in Table 2. Accordingly, the quercetin release data of both nanoparticles, either at pH 7.4 or pH 4.0, is best fitted to the Ritger–Peppas model compared to other models based on the *R*^2^ values. For example, the Ritger–peppas *R*^2^ value for MSN at pH 7.4 is 0.928 which higher than Higuchi (0.8918), First-order (0.8164), and Zero-order (0.7562). This indicates that the quercetin release mechanism follows the Ritger–Peppas model. This model describes of two cases that could be followed to determine the bioactive release mechanism. They are Fickian diffusion (Case I) and non-Fickian diffusion (including case II, anomalous transport, and super case II) which depends on the value of *n*. The *n* value of both nanoparticles at all pH conditions demonstrates *n* < 0.43 which also can be considered as a Fickian diffusion release mechanism for the case of polydispersed spherical materials^50^. As given in Table 2, the kinetic rate constant *k*_*RP*_ of MSN is higher than MSN-AmEA (except at pH 4.0 which the *k*_*RP*_ AmEA > MSN), indicating the diffusion rate of quercetin from MSN was faster than MSN-AmEA. This condition can be explained following these reasons: (i) texturally, the MSN has well-divine cylindrical pore structures and their size is bigger than MSN-AmEA. The pore size, as explained by Li et al.^51^, affects the release rate of entrapped drugs within nanoparticles in which the bigger pore size minimizes the boundaries of drugs released to the PBS environment hence increasing the release rate; (ii) The presence of defect structure along the pore channels of MSN-AmEA caused by the growing of siloxane network (Fig. 3d), might contribute in delaying of quercetin diffusion henceforth decreasing the diffusion rate constant; (iii) the electrostatic interaction of AmEA moieties with quercetin could slow down the diffusion rate. However, the kinetic release rate of MSN-AmEA at pH 4.0 was slightly higher than MSN, which confirmed the triggered release feature of this materials. The quercetin release rate under pH 7.4 was suppressed but it was elevated at pH 4.0 environment.

**Table 2:** The result of fitting the quercetin release data with the zero-order, first-order, Higuchi and Ritger–Peppas models.

Models	Parameters	Materials			
		MSN		MSN-AmEA	
		pH 7.4	pH 4.0	pH 7.4	pH 4.0
Zero-order	*k*_0_	0.91	0.51	0.08	0.71
	*R*^2^	0.7562	0.7851	0.929	0.8802
First-order	*k*_1_ × 10^−2^	1.45	0.63	0.08	0.96
	*R*^2^	0.8164	0.8101	0.929	0.8801
Higuchi	*k*_*H*_	7.04	3.93	0.62	5.31
	*R*^2^	0.8918	0.908	0.9899	0.9647
Ritger–Peppas	*k*_*RP*_	18.77	9.34	0.92	10.21
	*n*	0.23	0.27	0.39	0.32
	*R*^2^	0.9628	0.9599	0.9959	0.9864

### MEP and DFT analysis

**Figure 7:**
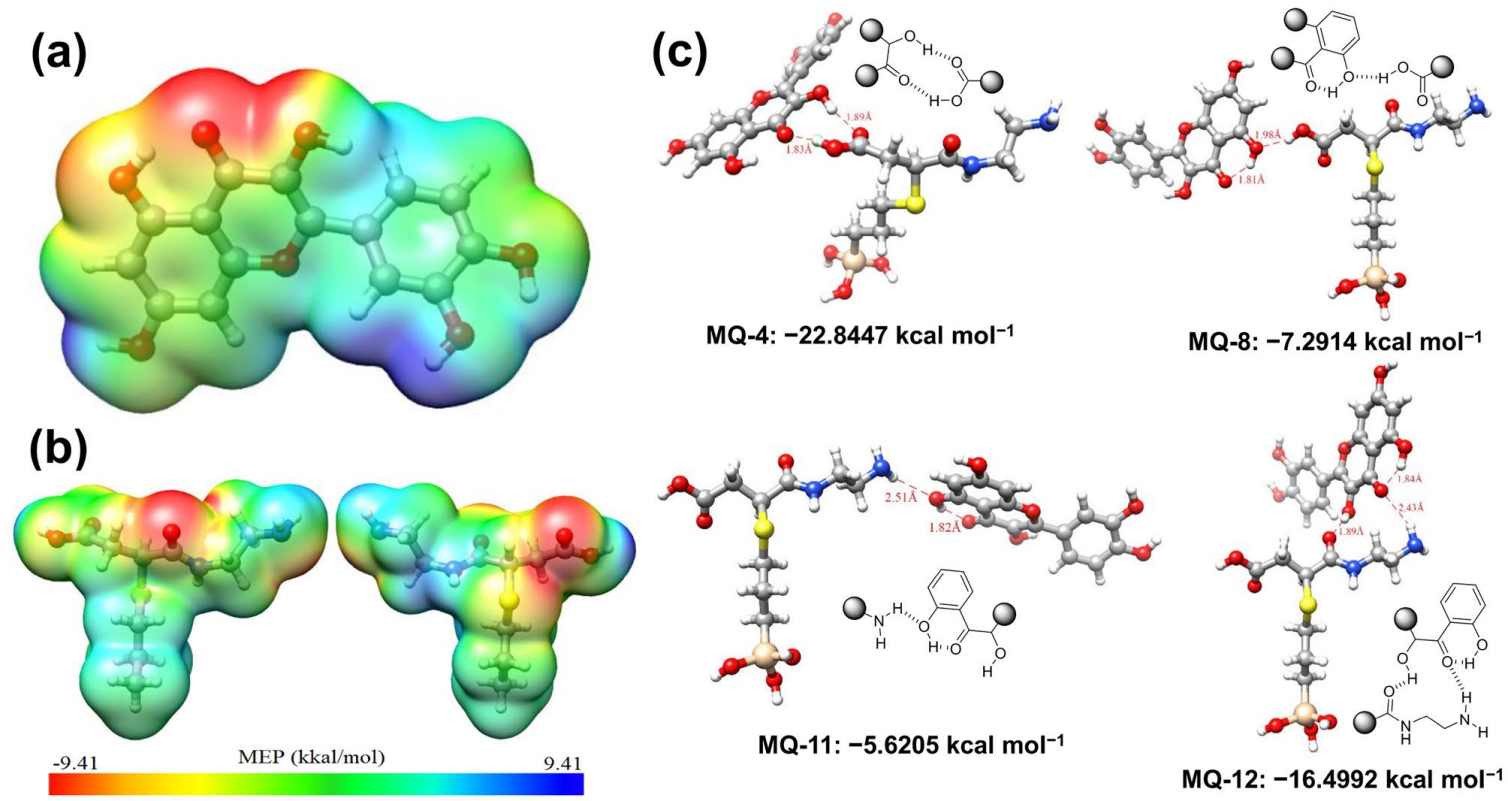
MEP analysis of (**a**) quercetin and (**b**) surface group moieties of MSN-AmEA (created by using UCSF Chimera version 1.12, https://www.cgl.ucsf.edu/chimera/index.html). (**c**) DFT model of interaction between quercetin and MSN-AmEA surface moieties at –COOH (MQ-4 and MQ-8) and –NH_2_ (MQ-11 and MQ-12) group position.

A computational approach, especially the calculation of molecular electrostatic potential (MEP) was performed in this report to study the localization of both nucleophilic and electrophilic sites. It can be used to evaluate intermolecular interaction^52^. This study is a convenient descriptor to determine the electronic density to predict the sites for nucleophilic-electrophilic attack sites^53^. In this study, the MEP of quercetin and AmEA moiety was calculated using B3LYP/6-31G(d,p) basis set in Gaussian09^54^. Visualization of the results was achieved using Chimera^37^ and Avogadro^55^. The result presented in Fig. 7a,b demonstrates a three-dimensional net electrostatic effect indicated by the red color which represents the partial electronegative part of molecules (nucleophilic sites), meanwhile, the blue region is the electrophilic site. The MEP map of quercetin (Fig. 7a and Supplementary Fig. S3a) indicates the possible strong electronegative charge density that lies on the oxygen atom at the C=O group (O4 position). Meanwhile, the strong electrophilic region (blue part) can be distinguished on the electropositive hydrogen atom at hydroxyl group O6 and O5 positions. Similar to the quercetin case, the MEP of AmEA moieties reveals two strong nucleophilic sites on the carbonyl group at O1 and O2 (Supplementary Fig. S3b). The positive electrostatic potential shown by the blue color of AmEA is represented by nitrogen atoms (N1 and N2, but more positive in N2) and also by hydrogen atoms of the carboxylate group at O3 position (which easily form H^+^ owing to low pKa ~ 5). Based on this calculation, it can be further used to electrostatically interact between quercetin and AmEA moieties through nucleophilic-electrophilic interactions.

We have initially modeled and calculated the AmEA-quercetin complexes with all possible hydrogen bonding (intra- or inter-molecular bonding) following the MEP data (electrophilic-nucleophilic electrostatic interaction) as depicted in Fig. 7c (the whole interaction can be found in Supplementary Figs. S5 and S6). The interaction energy and hydrogen bonding length of all complexes was depicted in Supplementary Table S2. Figure 7c shows the selected complex model having the both highest and lowest interaction energy either at the carboxylic group or the amines group position. As expected, a strong hydrogen bonding interaction occurred between the highly nucleophilic site of quercetin (C=O as electron-rich) with highly electrophilic sites of AmEA (primary amines and hydroxyl group as proton donor). For example, the interaction energy of the MQ-4 complex is − 22.8447 kcal mol^−1^ which is stronger than other complexes implying favorable and strong interaction among others. This is due to the double intermolecular hydrogen bonding between AmEA and quercetin molecules, consisting of C=O_2_∙∙∙H−O_2_ (1.89 Å, hydrogen bond angle, q = 152.26°) and O_3_−H∙∙∙O_4_=C (1.83 Å, q = 171.77°). In comparison, the MQ-8 has weak energy interaction, about − 7.2914 kcal mol^−1^, owing to only involving one intermolecular hydrogen O_3_−H∙∙∙O_3_−H (AmEA-Que) at the strong electrophilic site of AmEA and medium nucleophilic site of quercetin with the hydrogen bond length of 1.98 Å (q = 165.61). The amine groups also contribute to intermolecular hydrogen bonding to the quercetin molecules. Similar to the MQ-4 and MQ-8 complex cases, the interaction energy of AmEA at amines position with quercetin also depends on their position. Hydrogen interaction between the H atom at the N2 position of AmEA (strong electrophilic) with O_2_ of quercetin (medium nucleophilic) forming MQ-11 complex has interaction energy for about − 5.6205 kcal mol^−1^. On the other hand, the MQ-12 complex shows stronger interaction than the MQ-11 complex, which has − 16.4992 kcal mol^−1^, due to the AmEA providing two sites (both electrophilic and nucleophilic) for intermolecular hydrogen bonding interaction with quercetin (C=O_1_∙∙∙H−O_2_ and N_2_−H∙∙∙O_4_=C with hydrogen bond length of 1.89 Å and 2.43 Å, respectively).

Accordingly, this study implies a relatively very stable interaction of quercetin within MSN-AmEA nanoparticles supporting very low leaking release over neutral conditions as well as high loading capacity. Both carboxylates and amines functional groups of AmEA moieties play a vital role in preventing the premature release of quercetin under normal conditions. This is can be seen from the calculated interaction energy of 13 complex models optimized in this study. Therefore, introducing multifunctional groups onto the MSN surfaces is a beneficial strategy for overcoming drug delivery issues (loading capacity and premature release).

### Cellular uptake and viability test of nanoparticles against neuroblastoma cells

**Figure 8:**
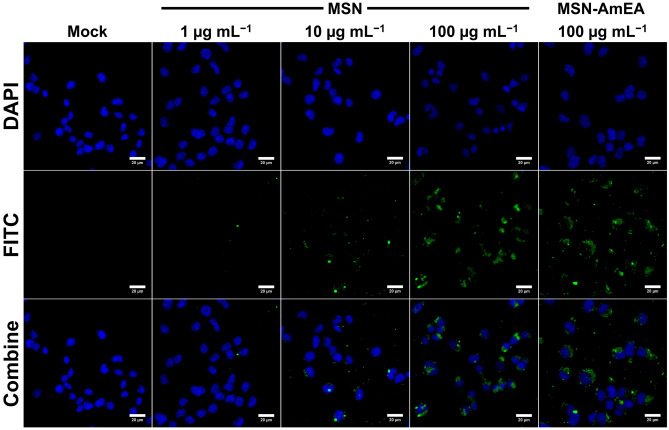
Fluorescence microscopic images of neuroblastoma cells treated by various concentration of FITC labelled MSN and MSN-AmEA.

We performed a preliminary in-vitro study of our nanoparticles to evaluate their cellular uptake and cytotoxicity against cancer cells. In this study, we used neuroblastma cancer cells as a model and treate them with MSN, MSN-AmEA, and quercetin loaded MSN-AmEA. Figure 8 shows the fluorescence microscopic image of neuroblastoma cancer cell treated with MSN at concentration of 1 µg mL^−1^ to 100 µg mL^−1^, and MSN-AmEA (100 µg mL^−1^). The data indicates that at concentration of 100 µg mL^−1^, both MSN and MSN-AmEA, capable to be taken by the cells. Therefore, we used this concentration for study the viability of MSN-AmEA nanoparticles containing quercetin.

**Figure 9:**
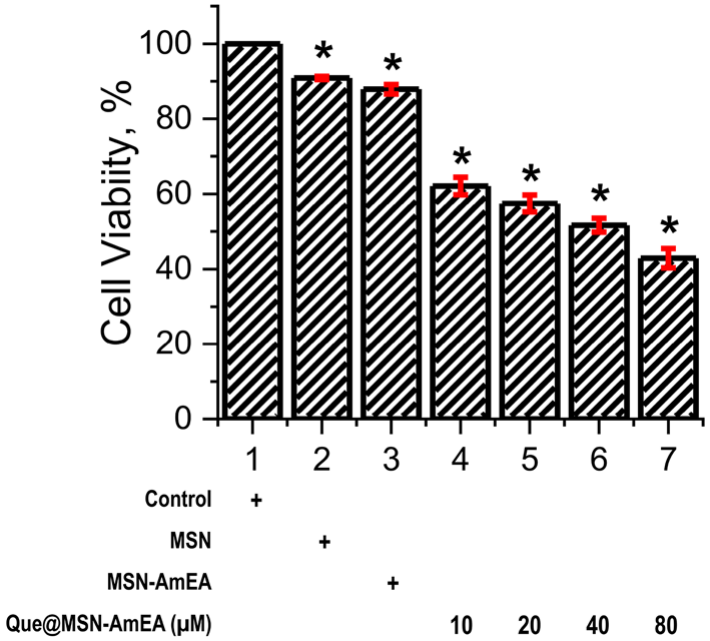
Viability test of neuroblastoma cells treated with MSN (100 µg mL^−1^), MSN-AmEA (100 µg mL^−1^), and Que@MSN-AmEA contained quercetin at concentration of 10, 20, 40 and 80 µM for 24 h.

Figure 9 reveals the cell viability of neuroblastoma treated with MSN, MSN-AmEA, and Que@MSN-AmEA. Both MSN and MSN-AmEA has high cellular viability, about 90.84% and 87.86%, respectively. This indicate that both of them has low toxicity. However, the cell viability of MSN-AmEA containing quercetin at concentration of 10, 20, 40 and 80 µM was decreased significantly. It was found that the cell viability of 10, 20 and 40 µM of Que@MSN-AmEA was above 50%, but it was 42.87% at a concentration of 80 µM. Based on this result, we confirmed the anticancer activity of quercetin loaded into MSN-AmEA.

## Conclusion

In this work, we successfully prepared MSN and were further chemically modified by introducing AmEA functional compound containing both amines and carboxylic groups through the thiol-ene click chemistry approach. The modification of nanoparticles was distinguished by simple characterization via functional group and thermogravimetry analysis by FTIR and TGA, respectively. The presence of functional compounds on the MSN nanostructure did not significantly alter the morphological and textural properties of its origin. However, the surface of MSN coated and partially intra-connected by a siloxane network decreased the BET surface area from 1038.53 m^2^ g^−1^ to 858.41 m^2^ g^−1^. In addition, owing to this phenomenon, the pore size of nanoparticles became smaller which suggests the functional compound attached alongside the MSN pore channels. Thanks to the AmEA, the effective adsorption capacity of nanoparticles toward quercetin compound was enhanced greatly by about 118%. Moreover, the controlled drug release behavior of Que@MSN-AmEA was achieved triggered by pH, in which more drugs were released under an artificial cancer environment than physiological conditions. The in-vitro cellular test also confirmed that the nanoparticles have good cellular uptake and cytotoxicity against neuroblastoma cancer cells. It was found that the MSN-AmEA containing 80 µM having cell viability about 42.87%. Therefore, the surface modification of MSN by AmEA through the thiol-ene ‘click chemistry’ strategy is proposed as one of the powerful pathways to preparing functional material for a smart drug delivery system. To improve its smart feature, a targeted molecule, for example, α_v_β_3_ integrin recognized peptide, will be introduced in our future work to the surfaces of the nanoparticles for enhancing the therapeutic efficacy. Hopefully, the combination of those functional compounds (AmEA and specific peptide for selectively binding to the overexpressed integrin receptor) leads to a powerful platform for cancer treatment.

## Supplementary Information

Supplementary Information.

## Data Availability

All data generated or analyzed during this study are included in this published article [and its supplementary information files].
