# Supplementary material for: Organically surface engineered mesoporous silica nanoparticles control the release of quercetin by pH stimuli
Source: Sci Rep. 2022 Nov 30;12:20661. doi: 10.1038/s41598-022-25095-4 (PMC9712501; doi:10.1038/s41598-022-25095-4)
Supplement: Supplementary file 1 — Supplementary Information. [file 41598_2022_25095_MOESM1_ESM.docx]

Supplementary Information

**Organically Surface Engineered Mesoporous Silica Nanoparticles Control the Release of Quercetin by pH Stimuli**

**Ozi Adi Saputra^1,2,3,4^, Windy Ayu Lestari^1^, Viardi Kurniasyah^5^, Witri Wahyu Lestari^5^, Takashi Sugiura^6^, Rino Rakhmata Mukti^7^, Ronny Martien^8^, Fajar Rakhman Wibowo^5,^***

^1^ Master Program of Chemistry, Faculty of Mathematics and Natural Sciences, Universitas Sebelas Maret, Jl. Ir. Sutami 36A, Surakarta, 57126, Indonesia.

^2^ Department of Chemical Engineering, Collage of Engineering, National Taiwan University, No. 1, Section 4, Roosevelt Rd, Da’an District, Taipei, 10617, Taiwan (Republic of China).

^3^ Institute of Chemistry, Academia Sinica, No. 128, Section 2, Academia Rd, Nangang District, Taipei, 11529, Taiwan (Republic of China)

^4^ Sustainable Chemical Science and Technology, Taiwan International Graduate Program, Academia Sinica, No. 128, Sec. 2, Academia Rd, Nangang District, Taipei, 11529, Taiwan (Republic of China).

^5^ Chemistry Department, Faculty of Mathematics and Natural Sciences, Universitas Sebelas Maret, Jl Ir. Sutami 36A, Surakarta, 57126, Indonesia

^6^ Department of Chemistry and Biomolecular Science, Faculty of Engineering, Gifu University, Gifu, 501-1193, Japan.

^7^ Division of Inorganic and Physical Chemistry, and Research Center for Nanosciences and Nanotechnology, Institut Teknologi Bandung, Jl. Ganesha no. 10 Bandung, 40132, Indonesia.

^8^ Faculty of Pharmacy, Universitas Gadjah Mada, Sekip Utara, Yogyakarta, 55281, Indonesia.

*Corresponding Email: [fajarrakhman@staff.uns.ac.id](mailto:fajarrakhman@staff.uns.ac.id)

Figures captions

Figure S1. The UV-Vis spectra and linear fitting graph of quercetin at various concentrations.

Figure S2. ^1^H-NMR of AmEA mixture compound showing Z form dominating the structure.

Figure S3. Zeta potential of MSN and MSN-AmEA measured in water.

Figure S4. Optimized structure and MEP of (a) quercetin and (b) AmEA moiety

Figure S5. Model DFT interaction of quercetin and AmEA moiety on the carboxylic group sites.

Figure S6. Model DFT interaction of quercetin and AmEA moiety on the amines group sites.

Figure S7. MEP of protonated Si-AmEA moieties at four position.

Table captions

Table S1. The summary output of regression statistics

Table S2. Bond length, bond angle, bond energy and interaction energy of AmEA-quercetin complexes.

Figure S1 shows the UV-Vis spectra of quercetin in different concentration ranging from 0.33 µM to 10.58 µM. The data was then fitted to obtain the regression curve as shown in the Figure S1.





Figure S1. The UV-Vis spectra and linear fitting graph of quercetin at various concentrations.

The output of regression fitting data from Figure S1 can be found in Table S1.

Table S1. The summary output of regression statistics

| *Regression Statistics* | |
| --- | --- |
| Multiple R | 0.999498957 |
| R Square | 0.998998164 |
| Adjusted R Square | 0.998797797 |
| Standard Error | 0.002728775 |
| Observations | 7 |

According to the regression fitting data, the LOQ and LOD can be calculated by using Equation S1 and S2.

$LOD=\frac{3.3\sigma}{S}$ S1

$LOQ=\frac{10\sigma}{S}$ S2

Here, σ is the standard error of the response and S is the slope obtained from the fitting curve. Therefore, the LOD and LOQ are found to be 0.4368 and 1.3236, respectively. Based on this data, all of the measured release concentration was above the LOQ, for example the MSN-AmEA (the lowest release percentage among all nanoparticles in both pH) has absorbance 0.073 which equal to 3.26 µM.

Figure S2 reveals the ^1^H-NMR of the AmEA compound measured with proton NMR 300 MHz. It can be seen that the compound was a mixture compound, however, the Z-isomer can be distinguished based on the proton signal in C=C having *J* value close to 10 as calculated from prediction data.


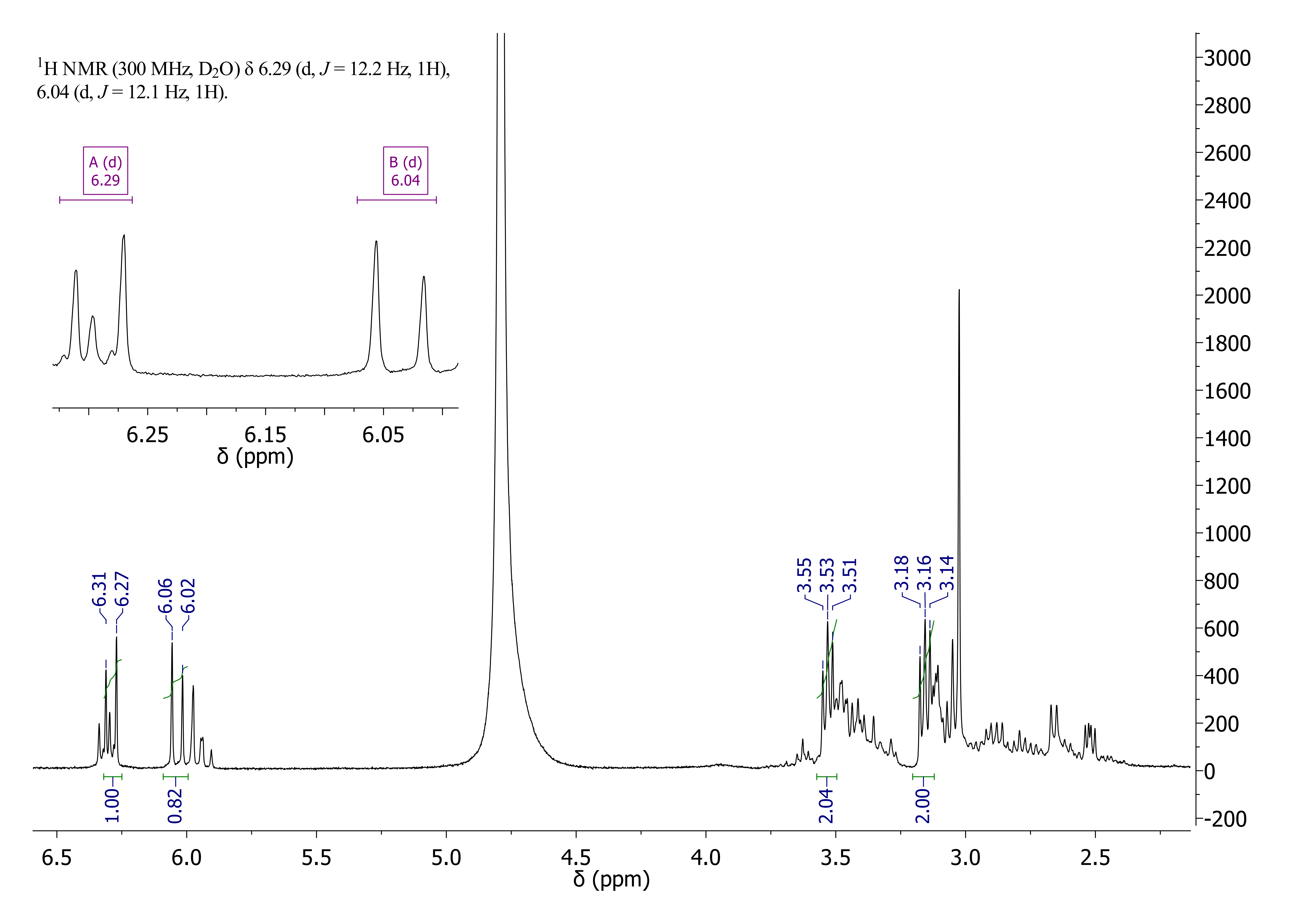


Figure S2. ^1^H-NMR of AmEA mixture compound showing Z form dominating the structure.

Figure S3 shows the zeta potential graph of MSN and MSN-AmEA which both of them measured in water. The MSN has zeta value of −27.3 mV, meanwhile the zeta potential of MSN-AmEA was −2.1 mV.


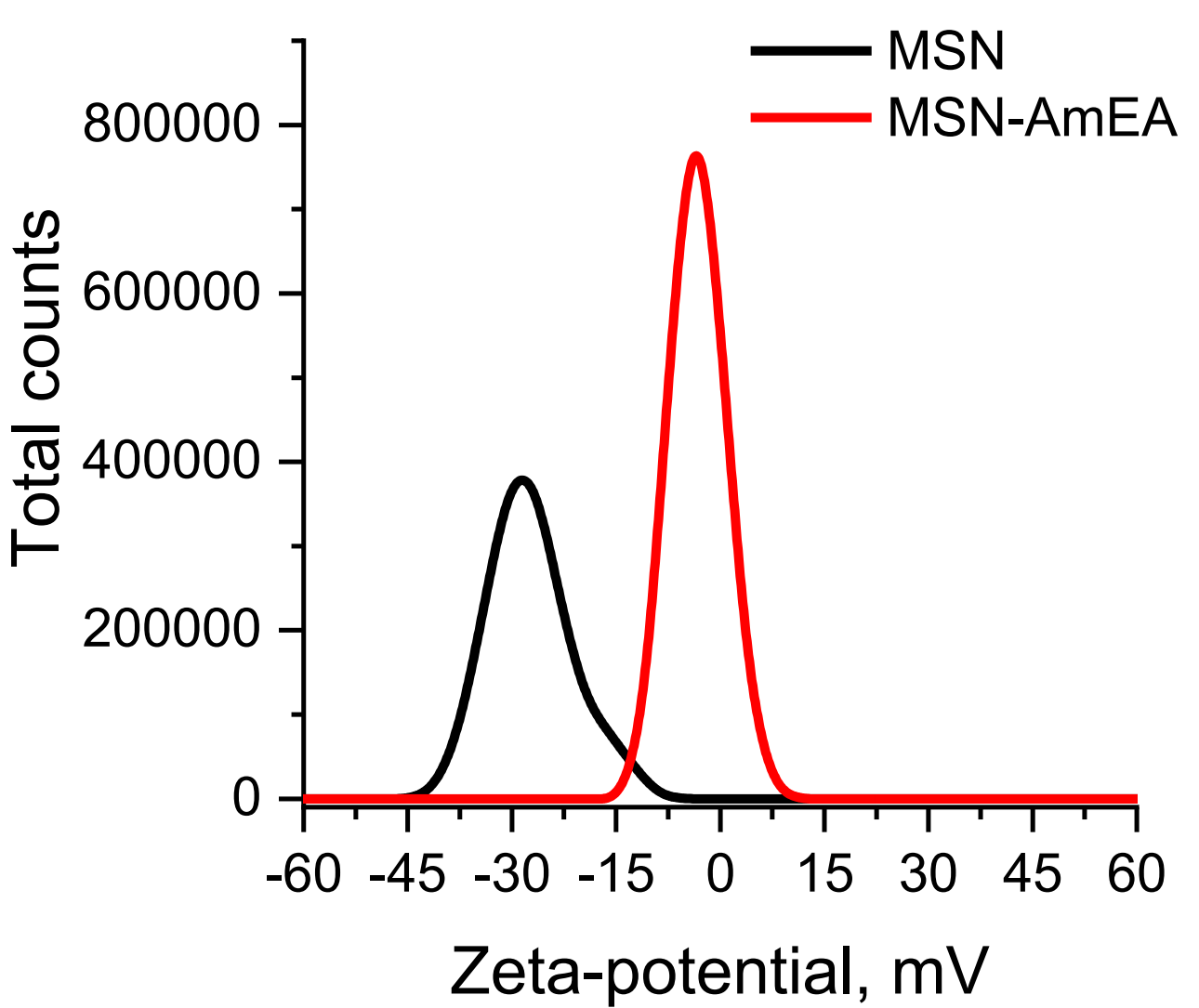


Figure S3. Zeta potential of MSN and MSN-AmEA measured in water.

Figure S4 depicts the optimized structure of quercetin and Si-AmEA with their respective MEP mapping. This data was used to predict the interaction between quercetin and Si-AmEA (nucleophilic-electrophilic) which the result can be found in the Figure S5, Figure S6, and Table S2.


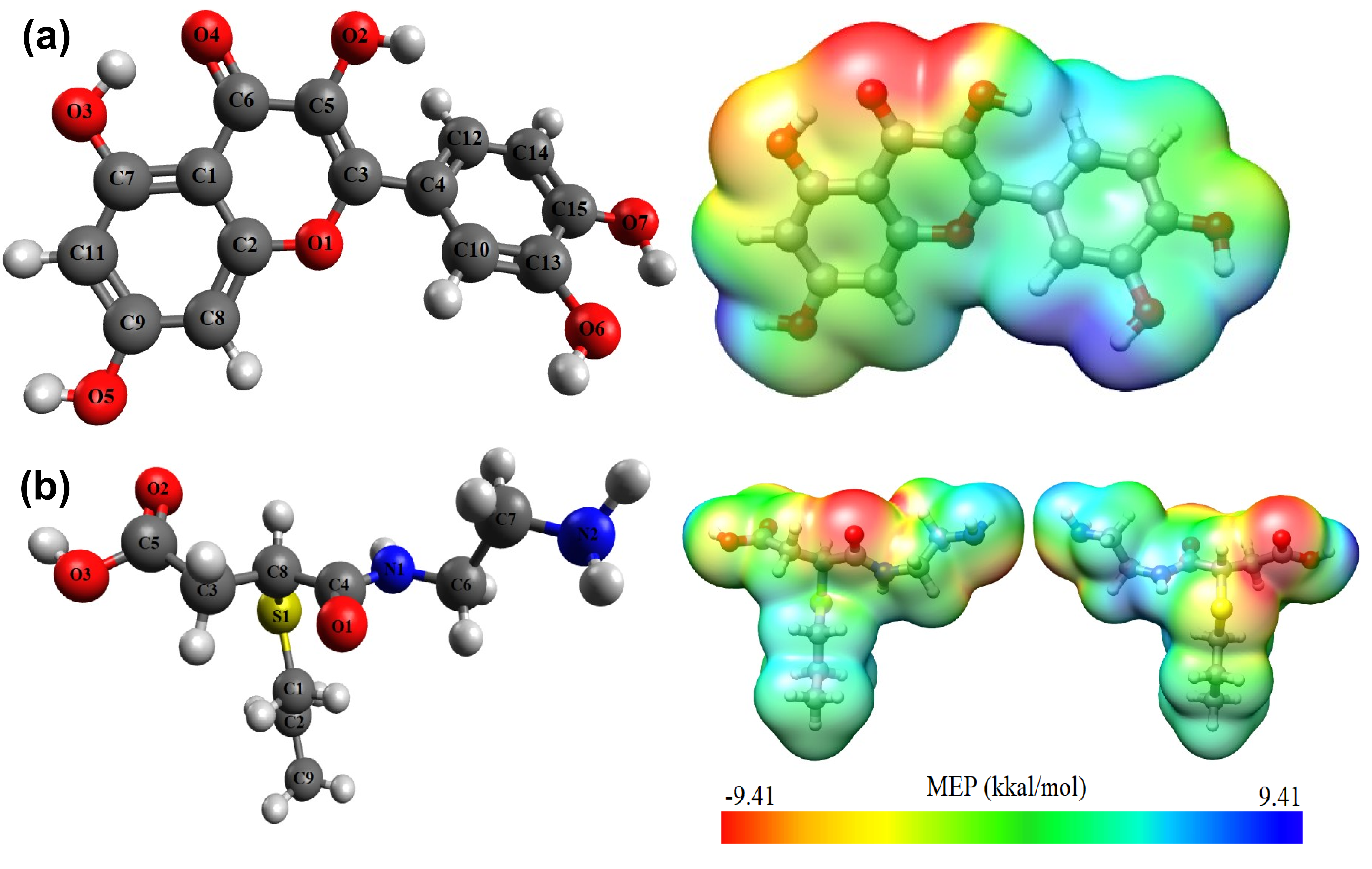


Figure S4. Optimized structure and MEP of (a) quercetin and (b) AmEA moiety


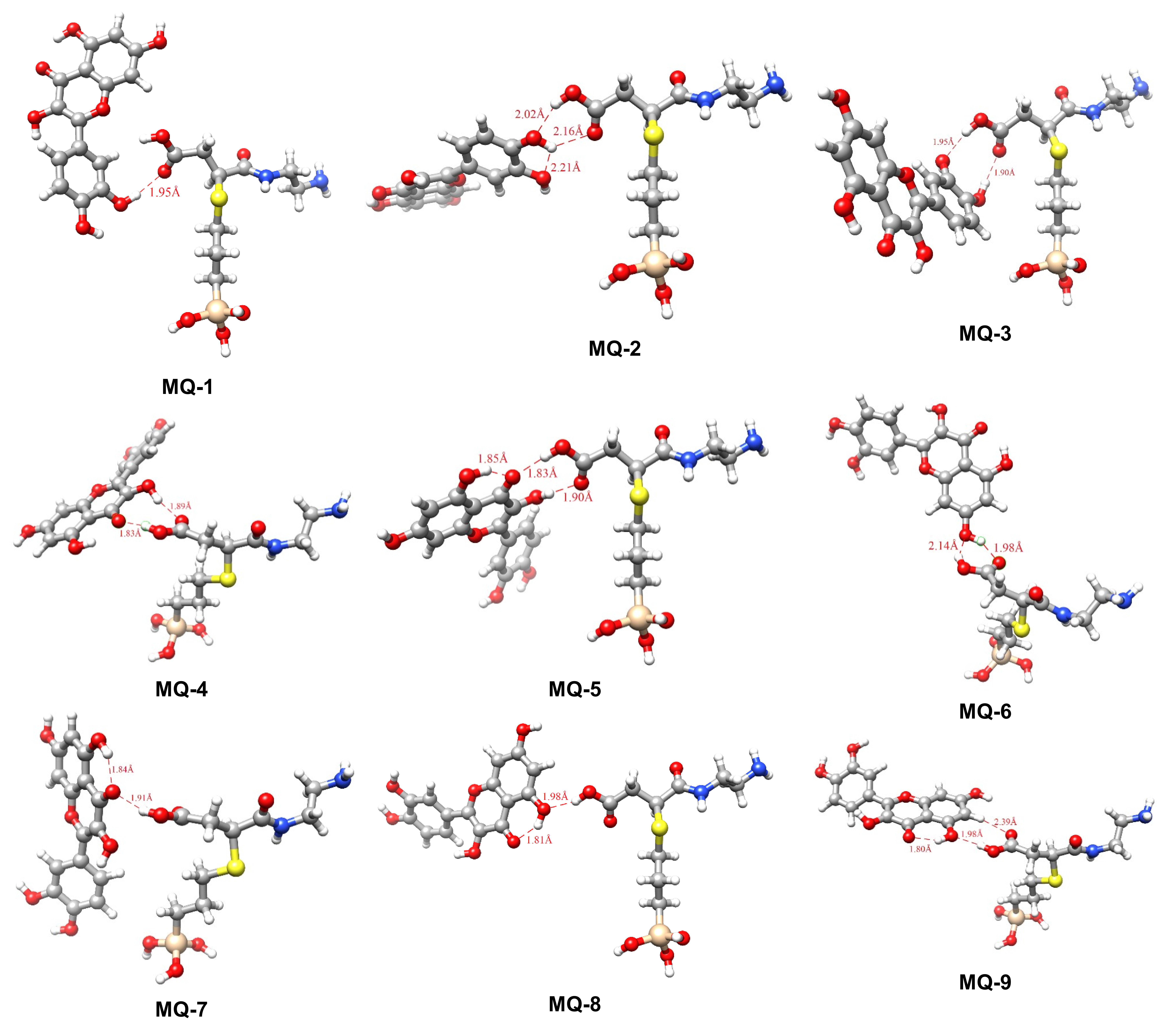


Figure S5. Model DFT interaction of quercetin and AmEA moiety on the carboxylic group sites.


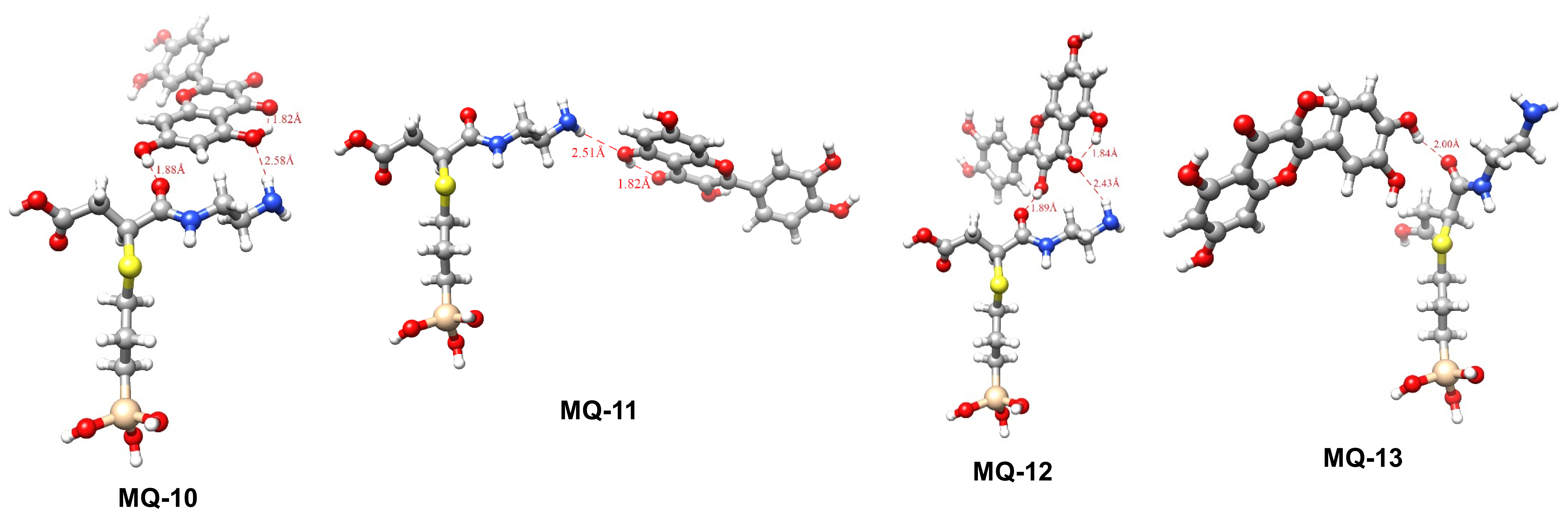


Figure S6. Model DFT interaction of quercetin and AmEA moiety on the amines group sites.

Table S2. Bond length, bond angle, bond energy and interaction energy of AmEA-quercetin complexes.

| Structure code | Interaction^*^ | Bond length  (Å) | Hydrogen bond angle, q  (°) | Bond Energy^**^  (Kcal mol^−1^) | Interaction Energy^***^  (Kcal mol^−1^) |
| --- | --- | --- | --- | --- | --- |
| MQ-1 | (M) C5=O2 --- (Q) H–O6 | 1.95027 | 166.84 | -8.6530 | -10.6882 |
| MQ-2 | (M) C5=O2 --- (Q) H–O7 | 2.16423 | 135.88 | -7.0363 | -9.3282 |
|  | (M) O3−H --- (Q) O7–H | 2.02021 | 153.69 |  |  |
| MQ-3 | (M) C5=O2 --- (Q) H–O7 | 1.90208 | 173.58 | -16.5026 | -21.1266 |
|  | (M) O3−H --- (Q) O6–H | 1.94661 | 166.87 |  |  |
| MQ-4 | (M) O3−H --- (Q) C6=O4 | 1.82906 | 171.77 | -15.0594 | -22.8447 |
|  | (M) C5=O2 --- (Q) H–O2 | 1.89217 | 152.26 |  |  |
| MQ-5 | (M) O3−H --- (Q) C6=O4 | 1.83209 | 172.77 | -16.4388 | -22.8038 |
|  | (M) C5=O2 --- (Q) H–O2 | 1.90163 | 151.83 |  |  |
| MQ-6 | (M) C5=O2 --- (Q) H–O5 | 1.97541 | 147.11 | -6.5885 | -9.7464 |
|  | (M) O3−H --- (Q) O5–H | 2.14159 | 151.36 |  |  |
| MQ-7 | (M) O3−H --- (Q) C6=O4 | 1.90742 | 164.27 | -8.2048 | -10.6550 |
| MQ-8 | (M) O3−H --- (Q) O3−H | 1.97784 | 165.61 | -5.1311 | -7.2914 |
| MQ-9 | (M) O3−H --- (Q) O3−H | 1.98028 | 169.99 | -5.8883 | -9.6227 |
|  | (M) C5=O2 --- (Q) H–C11 | 2.39124 | 160.69 |  |  |
| MQ-10 | (M) N2−H --- (Q) O3−H | 2.57974 | 162.26 | -10.7010 | -12.7760 |
|  | (M) C4=O1 --- (Q) H–O5 | 1.88407 | 174.28 |  |  |
| MQ-11 | (M) N2−H --- (Q) O3−H | 2.50543 | 152.72 | -3.8102 | -5.6205 |
| MQ-12 | (M) N2−H --- (Q) O4–C6 | 2.43082 | 146.85 | -13.4215 | -16.4992 |
|  | (M) C4=O1 --- (Q) H–O2 | 1.88302 | 142.03 |  |  |
| MQ-13 | (M) C4=O1 --- (Q) H–O7 | 1.99769 | 160.43 | -4.9113 | -7.2306 |
| ^*^ M and Q indicate chemical groups on surface moieties and quercetin, respectively.  ^**^ Bond energy was calculated based on HF method.  ^***^ Interaction energy was calculated based on DFT method. | | | | | |

Figure S7 indicates the change in MEP energy ranges after being protonated by H^+^. The original Si-AmEA has a MEP range of -9.41 kcal/mol to 9.41 kcal/mol, and become 31.38 kcal/mol to 94.13 kcal/mol.


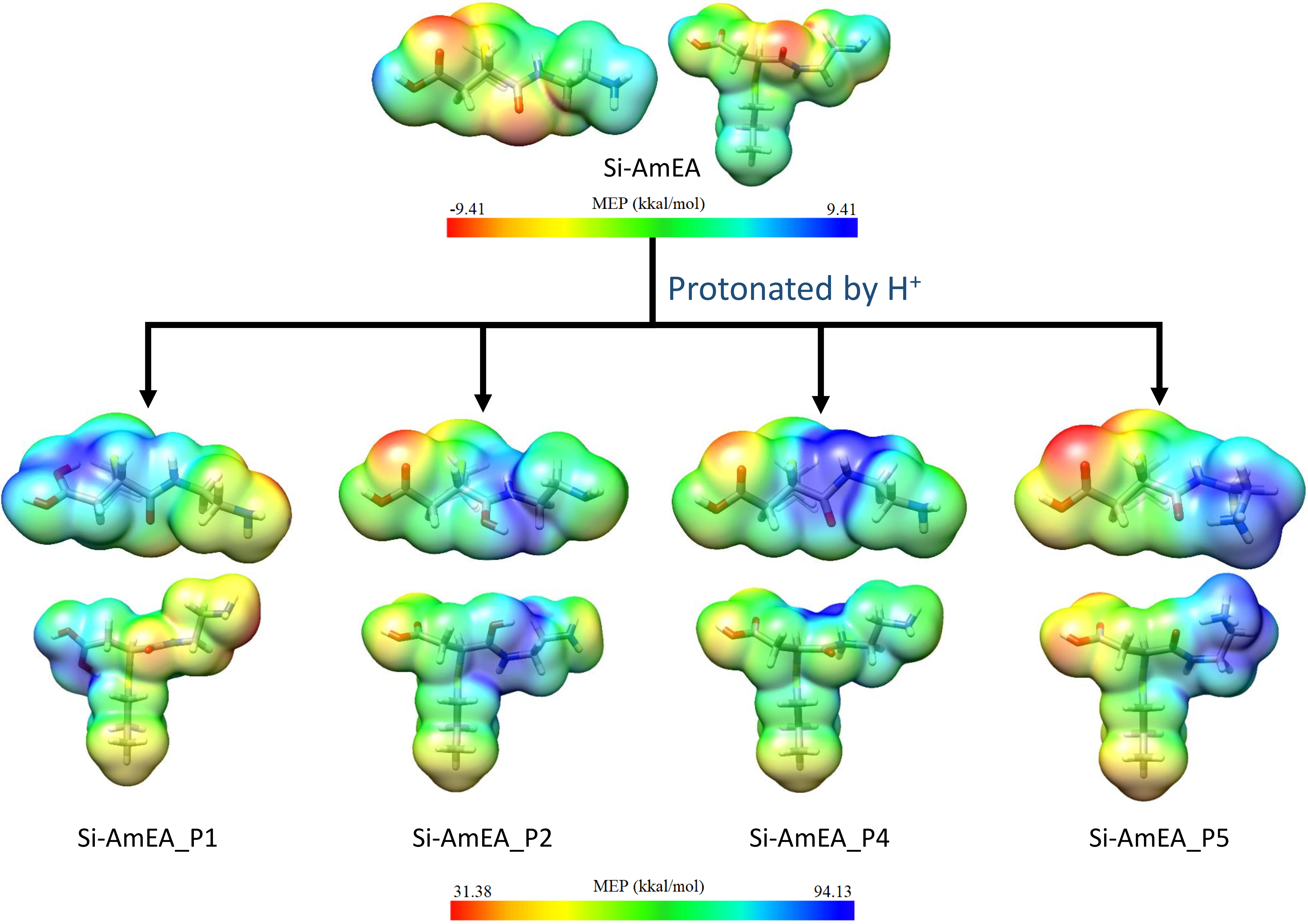


Figure S7. MEP of protonated Si-AmEA moieties at four position.
